# Supplementary material for: Hyperfine-Resolved Spectroscopy of Dysprosium Monoxide (DyO)
Source: J Phys Chem A. 2025 Dec 29;130(2):513–21. doi: 10.1021/acs.jpca.5c07797 (PMC12814554; doi:10.1021/acs.jpca.5c07797)
Supplement: Supplementary file 1 [file jp5c07797_si_001.pdf]

Supporting Information
"Hyperfine-Resolved Spectroscopy of Dysprosium Monoxide (DyO)"

by Zack D. Lasner, Aidan T. Ohl, Nicole M. Albright, Kendall L. Rice, Charlene Peng, Lan Cheng, John M. Doyle, and Benjamin L. Augenbraun

Table S1. Observed and calculated transition wavenumbers (in cm^{-1}) of the ^{161}DyO [17.1]7-X8 (0,0) band. Transitions are labeled by $\Delta J_{\Delta F}(J)$ and F, where J are F are the lower-state rotational and hyperfine quantum numbers, respectively.

Table S2. Observed and calculated transition wavenumbers (in cm^{-1}) of the ^{161}DyO [17.1]7-X8 (0,1) band. Transitions are labeled by $\Delta J_{\Delta F}(J)$ and F, where J are F are the lower-state rotational and hyperfine quantum numbers, respectively.

Table S3. Observed and calculated transition wavenumbers (in cm^{-1}) of the ^{163}DyO [17.1]7-X8 (0,0) band. Transitions are labeled by $\Delta J_{\Delta F}(J)$ and F, where J are F are the lower-state rotational and hyperfine quantum numbers, respectively.

Table S4. Observed and calculated transition wavenumbers (in cm^{-1}) of the ^{163}DyO [17.1]7-X8 (0,1) band. Transitions are labeled by $\Delta J_{\Delta F}(J)$ and F, where J are F are the lower-state rotational and hyperfine quantum numbers, respectively.

Table S5. Parameter correlation matrix for the ^{161}DyO fit.

Table S6. Parameter correlation matrix for the ^{163}DyO fit.

Table S1. Observed and calculated transition wavenumbers (in cm^{-1}) of the ^{161}DyO [17.1]7-X8 (0,0) band. Transitions are labeled by $\Delta J_{\Delta F}(J)$ and F, where J are F are the lower-state rotational and hyperfine quantum numbers, respectively.

Transition	F	Obs.	Calc.	Obs. - Calc.
Pp(8)	5.5	17056.0969	17056.1012	0.0044
	6.5	17056.1142	17056.1163	0.0021
	7.5	17056.1344	17056.1351	0.0008
	8.5	17056.1611	17056.1588	-0.0022
	9.5	17056.1908	17056.1887	-0.0021
	10.5	17056.2275	17056.2261	-0.0013
Pp(9)	6.5	17054.0486	17054.0485	-0.0001
	7.5	17054.0640	17054.0639	0.0000
	8.5	17054.0818	17054.0824	0.0006
	9.5	17054.1040	17054.1044	0.0004
	10.5	17054.1303	17054.1308	0.0005
	11.5	17054.1622	17054.1624	0.0002
Pq(9)	10.5	17054.1126	17054.1119	-0.0007
Qp(8)	9.5	17060.6187	17060.6181	-0.0006
	10.5	17060.6538	17060.6543	0.0005
Qq(8)	5.5	17060.4959	17060.4958	-0.0001
	6.5	17060.5151	17060.5147	-0.0004
	7.5	17060.5384	17060.5376	-0.0008
	8.5	17060.5649	17060.5650	0.0001
	9.5	17060.5976	17060.5974	-0.0002
	10.5	17060.6362	17060.6355	-0.0008
Qr(8)	8.5	17060.5446	17060.5443	-0.0003
Qq(9)	6.5	17059.1845	17059.1810	-0.0035
	7.5	17059.1998	17059.1993	-0.0005
	8.5	17059.2207	17059.2208	0.0001
	9.5	17059.2442	17059.2456	0.0014
	10.5	17059.2727	17059.2739	0.0012
	11.5	17059.3057	17059.3062	0.0005
Rr(8)	5.5	17065.6296	17065.6282	-0.0014
	6.5	17065.6516	17065.6501	-0.0015
	7.5	17065.6760	17065.6760	0.0000
	8.5	17065.7048	17065.7061	0.0014
	9.5	17065.7387	17065.7405	0.0018
	10.5	17065.7786	17065.7793	0.0007

Table S2. Observed and calculated transition wavenumbers (in cm^{-1}) of the ^{161}DyO [17.1]7-X8 (0,1) band. Transitions are labeled by $\Delta J_{\Delta F}(J)$ and F, where J are F are the lower-state rotational and hyperfine quantum numbers, respectively.

Transition	F	Obs.	Calc.	Obs. - Calc.
Pp(8)	5.5	16213.7193	16213.7223	0.0030
	6.5	16213.7372	16213.7388	0.0016
	7.5	16213.7584	16213.7587	0.0003
	8.5	16213.7836	16213.7829	-0.0007
	9.5	16213.8141	16213.8122	-0.0019
	10.5	16213.8500	16213.8480	-0.0020
Pq(8)	5.5	16213.6974	16213.6984	0.0010
	6.5	16213.7134	16213.7136	0.0002
	7.5	16213.7344	16213.7336	-0.0008
	8.5	16213.7609	16213.7596	-0.0013
	9.5	16213.7938	16213.7928	-0.0010
Pp(9)	6.5	16211.6972	16211.6969	-0.0003
	7.5	16211.7127	16211.7133	0.0006
	8.5	16211.7311	16211.7325	0.0014
	9.5	16211.7531	16211.7548	0.0017
	10.5	16211.7795	16211.7809	0.0014
	11.5	16211.8113	16211.8113	0.0000
Pq(9)	6.5	16211.6762	16211.6768	0.0006
	7.5	16211.6904	16211.6921	0.0017
	8.5	16211.7089	16211.7110	0.0022
	9.5	16211.7311	16211.7341	0.0030
	10.5	16211.7612	16211.7620	0.0008
Qp(8)	7.5	16218.1838	16218.1824	-0.0014
	8.5	16218.2105	16218.2105	0.0000
	9.5	16218.2417	16218.2417	0.0000
	10.5	16218.2766	16218.2762	-0.0004
Qq(8)	5.5	16218.1200	16218.1168	-0.0032
	6.5	16218.1394	16218.1372	-0.0022
	7.5	16218.1619	16218.1612	-0.0007
	8.5	16218.1888	16218.1890	0.0002
	9.5	16218.2209	16218.2210	0.0001
	10.5	16218.2582	16218.2573	-0.0009
Qr(8)	5.5	16218.0991	16218.0967	-0.0024
	6.5	16218.1165	16218.1160	-0.0005
	7.5	16218.1394	16218.1397	0.0004

	8.5	16218.1682	16218.1683	0.0001
	9.5	16218.2026	16218.2021	-0.0005
Qp(9)	7.5	16216.8696	16216.8659	-0.0038
	8.5	16216.8916	16216.8891	-0.0025
	9.5	16216.9150	16216.9147	-0.0003
	10.5	16216.9423	16216.9428	0.0005
	11.5	16216.9723	16216.9733	0.0010
Qq(9)	6.5	16216.8329	16216.8293	-0.0036
	7.5	16216.8509	16216.8487	-0.0022
	8.5	16216.8709	16216.8709	0.0000
	9.5	16216.8950	16216.8960	0.0010
	10.5	16216.9230	16216.9240	0.0010
	11.5	16216.9557	16216.9551	-0.0006
Qr(9)	6.5	16216.8169	16216.8122	-0.0047
	7.5	16216.8296	16216.8306	0.0010
	8.5	16216.8556	16216.8522	-0.0034
	9.5	16216.8749	16216.8773	0.0024
	10.5	16216.9050	16216.9059	0.0009
Rr(8)	6.5	16223.2754	16223.2726	-0.0028
	7.5	16223.2994	16223.2996	0.0002
	8.5	16223.3281	16223.3302	0.0021
	9.5	16223.3628	16223.3641	0.0013
	10.5	16223.4015	16223.4011	-0.0004

Table S3. Observed and calculated transition wavenumbers (in cm^{-1}) of the ^{163}DyO [17.1]7-X8 (0,0) band. Transitions are labeled by $\Delta J_{\Delta F}(J)$ and F, where J are F are the lower-state rotational and hyperfine quantum numbers, respectively.

Transition	F	Obs.	Calc.	Obs. - Calc.
Pp(8)	5.5	17056.2204	17056.2191	-0.0014
	6.5	17056.1868	17056.1858	-0.0009
	7.5	17056.1526	17056.1500	-0.0026
	8.5	17056.1142	17056.1127	-0.0015
	9.5	17056.0755	17056.0753	-0.0002
	10.5	17056.0341	17056.0393	0.0052
Pq(8)	5.5	17056.2371	17056.2353	-0.0018
	6.5	17056.2099	17056.2080	-0.0020
	7.5	17056.1797	17056.1796	-0.0001
	8.5	17056.1526	17056.1516	-0.0010
Pp(9)	6.5	17054.1557	17054.1551	-0.0006
	7.5	17054.1255	17054.1258	0.0003
	8.5	17054.0936	17054.0942	0.0006
	9.5	17054.0608	17054.0611	0.0003
	10.5	17054.0276	17054.0271	-0.0005
	11.5	17053.9932	17053.9931	-0.0001
Pq(9)	6.5	17054.1709	17054.1719	0.0011
	7.5	17054.1467	17054.1472	0.0005
	8.5	17054.1205	17054.1211	0.0006
	10.5	17054.0698	17054.0680	-0.0018
Qp(8)	9.5	17060.4429	17060.4442	0.0014
Qq(8)	5.5	17060.6300	17060.6297	-0.0003
	6.5	17060.5970	17060.5971	0.0001
	7.5	17060.5601	17060.5605	0.0004
	8.5	17060.5206	17060.5204	-0.0002
	9.5	17060.4769	17060.4775	0.0006
	10.5	17060.4325	17060.4323	-0.0002
Qr(8)	5.5	17060.6463	17060.6465	0.0002
	7.5	17060.5876	17060.5873	-0.0003
	8.5	17060.5543	17060.5537	-0.0005
Qp(9)	10.5	17059.1333	17059.1337	0.0004
Qq(9)	6.5	17059.2977	17059.2982	0.0004
	7.5	17059.2672	17059.2690	0.0018
	8.5	17059.2342	17059.2365	0.0023
	9.5	17059.2007	17059.2010	0.0003

	10.5	17059.1638	17059.1627	-0.0011
	11.5	17059.1265	17059.1220	-0.0045
Rr(8)	5.5	17065.7719	17065.7728	0.0009
	6.5	17065.7384	17065.7403	0.0019
	7.5	17065.7010	17065.7028	0.0018
	8.5	17065.6590	17065.6603	0.0013
	9.5	17065.6138	17065.6131	-0.0007

Table S4. Observed and calculated transition wavenumbers (in cm^{-1}) of the ^{163}DyO [17.1]7-X8 (0,1) band. Transitions are labeled by $\Delta J_{\Delta F}(J)$ and F, where J are F are the lower-state rotational and hyperfine quantum numbers, respectively.

Transition	F	Obs.	Calc.	Obs. - Calc.
Pp(8)	5.5	16214.3080	16214.3069	-0.0011
	6.5	16214.2755	16214.2737	-0.0018
	7.5	16214.2396	16214.2380	-0.0016
	8.5	16214.2014	16214.2008	-0.0006
	9.5	16214.1619	16214.1635	0.0016
	10.5	16214.1215	16214.1274	0.0059
Pq(8)	5.5	16214.3245	16214.3231	-0.0014
	6.5	16214.2974	16214.2959	-0.0015
	7.5	16214.2687	16214.2676	-0.0011
	8.5	16214.2363	16214.2396	0.0034
	9.5	16214.2090	16214.2135	0.0045
Pp(9)	6.5	16212.2695	16212.2694	-0.0001
	7.5	16212.2398	16212.2402	0.0004
	8.5	16212.2082	16212.2087	0.0005
	9.5	16212.1749	16212.1756	0.0007
	10.5	16212.1411	16212.1417	0.0006
	11.5	16212.1076	16212.1077	0.0001
Pq(9)	6.5	16212.2855	16212.2862	0.0007
	7.5	16212.2606	16212.2615	0.0010
	8.5	16212.2351	16212.2355	0.0004
	9.5	16212.2082	16212.2089	0.0007
	10.5	16212.1837	16212.1825	-0.0012
Qp(8)	6.5	16218.6693	16218.6682	-0.0011
	7.5	16218.6282	16218.6271	-0.0011
	8.5	16218.5818	16218.5817	-0.0001
	9.5	16218.5321	16218.5323	0.0002
	10.5	16218.4784	16218.4796	0.0012
Qq(8)	5.5	16218.7182	16218.7175	-0.0007
	6.5	16218.6852	16218.6850	-0.0002
	7.5	16218.6488	16218.6485	-0.0003
	8.5	16218.6091	16218.6085	-0.0006
	9.5	16218.5652	16218.5656	0.0004
	10.5	16218.5201	16218.5204	0.0003
Qr(8)	5.5	16218.7331	16218.7343	0.0012
	6.5	16218.7068	16218.7064	-0.0004

	7.5	16218.6755	16218.6753	-0.0002
	8.5	16218.6429	16218.6418	-0.0011
	9.5	16218.6091	16218.6065	-0.0027
Qp(9)	7.5	16217.3666	16217.3664	-0.0002
	8.5	16217.3293	16217.3305	0.0012
	9.5	16217.2899	16217.2910	0.0012
	10.5	16217.2479	16217.2483	0.0004
	11.5	16217.2025	16217.2024	-0.0001
Qq(9)	6.5	16217.4126	16217.4125	-0.0001
	7.5	16217.3826	16217.3834	0.0008
	8.5	16217.3499	16217.3510	0.0011
	9.5	16217.3152	16217.3155	0.0003
	10.5	16217.2779	16217.2773	-0.0006
	11.5	16217.2412	16217.2366	-0.0046
Qr(9)	6.5	16217.4280	16217.4294	0.0014
	7.5	16217.4026	16217.4038	0.0012
	8.5	16217.3753	16217.3754	0.0001
	9.5	16217.3466	16217.3445	-0.0021
	10.5	16217.3152	16217.3114	-0.0038
Rr(8)	5.5	16223.8598	16223.8606	0.0008
	6.5	16223.8271	16223.8282	0.0011
	7.5	16223.7897	16223.7908	0.0011
	8.5	16223.7484	16223.7484	0.0000
	9.5	16223.7023	16223.7012	-0.0011
	10.5	16223.6523	16223.6493	-0.0030

Table S5. Parameter correlation matrix for the ^{161}DyO fit.

		[17.1]7(v=0)					X8(v=0)			X8(v=1)			
		T_v	B	h	eQq_0	D	B	h	eQq0	T_v	B	h	eQq_0
[17.1]7 (v=0)	T_v	1.00											
	B	-0.74	1.00										
	h	-0.03	0.05	1.00									
	eQq_0	0.02	0.03	-0.25	1.00								
	D	-0.68	0.97	0.01	0.04	1.00							
X8 (v=0)	B	0.37	0.18	0.07	0.08	0.13	1.00						
	h	-0.06	0.07	0.87	-0.25	0.04	0.07	1.00					
	eQq_0	0.03	0.00	-0.23	0.65	0.01	0.05	-0.17	1.00				
X8 (v=1)	T_v	0.51	0.00	0.08	0.01	0.03	0.62	0.06	0.00	1.00			
	B	-0.21	0.18	0.04	0.03	0.07	0.11	0.04	0.02	-0.53	1.00		
	h	-0.04	0.06	0.88	-0.16	0.02	0.08	0.76	-0.17	0.10	0.06	1.00	
	eQq_0	0.03	0.01	-0.26	0.63	0.02	0.03	-0.25	0.42	0.05	-0.02	-0.12	1.00

Table S6. Parameter correlation matrix for the ^{163}DyO fit.

		[17.1]7(v=0)					X8(v=0)			X8(v=1)			
		T_v	B	h	eQq_0	D	B	h	eQq_0	T_v	B	h	eQq_0
[17.1]7 (v=0)	T_v	1.00											
	B	-0.71	1.00										
	h	-0.14	0.05	1.00									
	eQq_0	0.09	-0.04	-0.06	1.00								
	D	-0.65	0.97	0.00	-0.02	1.00							
X8 (v=0)	B	0.35	0.24	0.02	0.02	0.18	1.00						
	h	-0.22	0.05	0.82	0.04	0.00	-0.04	1.00					
	eQq_0	-0.01	-0.03	-0.09	0.64	-0.01	-0.05	0.15	1.00				
X8 (v=1)	T_v	0.47	0.09	-0.04	0.04	0.09	0.63	-0.16	-0.06	1.00			
	B	-0.17	0.15	0.09	-0.03	0.04	0.12	0.08	-0.03	-0.50	1.00		
	h	-0.13	0.05	0.87	-0.01	0.00	0.02	0.72	-0.05	0.01	0.08	1.00	
	eQq_0	0.07	-0.03	-0.13	0.59	-0.02	0.01	-0.06	0.38	0.06	-0.02	0.01	1.00
